# Risk factors of incontinence-associated dermatitis among critically ill patients: A systematic review and meta-analysis

**DOI:** 10.3389/fmed.2023.1146697

**Published:** 2023-04-11

**Authors:** Hongzhan Jiang, Jiali Shen, Huihui Lin, Qiuqin Xu, Yuanchan Li, Lijuan Chen

**Affiliations:** ^1^Nursing College, Fujian University of Traditional Chinese Medicine, Fuzhou, China; ^2^Department of General Surgery, Zhongshan Hospital of Xiamen University, School of Medicine, Xiamen, China

**Keywords:** incontinence-associated dermatitis, critical care, risk factors, systematic review, meta-analysis

## Abstract

**Objectives:**

Incontinence-associated dermatitis (IAD) is increasingly found among critically ill patients, but the risk factors for IAD in these patients are currently unclear. The purpose of this meta-analysis was to identify the risk factors of IAD in critically ill patients.

**Methods:**

Web of Science, PubMed, EMBASE, and Cochrane Library were systemically searched until July 2022. The studies were selected based on inclusion criteria, and data were independently extracted by two researchers. The Newcastle-Ottawa Scale (NOS) was used to assess the quality of the included studies. Odds ratios (ORs) and their associated 95% confidence intervals (CIs) were used to identify significant differences in the risk factors. The *I*^2^ test was used to estimate the heterogeneity of studies, and Egger's test was used to assess the potential publication bias.

**Results:**

A total of 7 studies enrolling 1,238 recipients were included in the meta-analysis. Age ≥ 60 (OR = 2.18, 95% CI: 1.38~3.42), female sex (OR = 1.76, 95% CI: 1.32~2.34), dialysis (OR = 2.67, 95% CI: 1.51~4.73), fever (OR = 1.55, 95% CI: 1.03~2.33), vasoactive agent (OR = 2.35, 95% CI: 1.45~3.80), PAT score ≥ 7 (OR = 5.23, 95% CI: 3.15~8.99), frequency of bowel movement > 3times/d (OR = 5.33, 95% CI: 3.19~8.93), and liquid stool (OR = 2.61, 95% CI: 1.56~4.38) were the risk factors of IAD among critically ill patients.

**Conclusions:**

Many risk factors are related to IAD among critically ill patients. Nursing staff should pay more attention to evaluating the risk of IAD and enhance the care of high-risk groups.

## Introduction

The term moisture-associated skin damage (MASD) is defined as inflammation and erosion of the skin caused by prolonged exposure to moisture and its contents, including urine, stool, perspiration, wound exudate, mucus, or saliva. MASD encompasses the four forms of moisture-associated skin damage, which include: (1) incontinence-associated dermatitis (IAD); (2) intertriginous dermatitis (ITD), also called intertrigo; (3) peristomal moisture-associated dermatitis; and (4) periwound moisture-associated dermatitis (1). IAD is a serious health problem on a global scale (2). It is common skin damage that is reported in clinical settings, especially when hospitalized in intensive care units (3). The main symptoms of IAD include skin impregnation, erythema reddish, desquamation, or erosion, mostly in the perineum (4). IAD sufferers frequently feel uncomfortable, burning sensation, itching, or pricking in the area of infected skin.

Nursing for critically ill individuals presents a significant challenge in maintaining skin integrity. The skin, the greatest human organ, not only serves as a highly efficient quality indicator but is also closely related to the patient's safety. Critically ill patients due to diminished cognitive and sensory functioning, most of the patients are not able to adequately react to pain and irritation caused by incontinence. Research has increasingly indicated that critically ill patients are at a high risk of IAD (5). Gray et al. (6) reported that 45.7% of individuals with any type of incontinence developed IAD. 73% were acquired when a patient was hospitalized, and more than 25% were present at admission. The incidence of IAD among critically ill patients ranges from 26.2 to 64% (7–9), which is higher than that of other inpatients. In addition, IAD is a recognized risk factor for pressure injury. Compared to people without incontinence, those with incontinence, particularly dual incontinence, had a 1.92~4.99 times higher risk of developing a facility-acquired pressure injury (10). IAD may lengthen the patient's hospital stay, drive up medical costs and nursing staff burden, and degrade the patients' quality of life (11). Therefore, reviewing the risk factors for IAD in critically ill patients is essential to creating a clinical foundation for effective nursing interventions for IAD management and prevention.

Many studies have reported the risk factors for IAD in critically ill patients, including diminished cognitive awareness, frequent liquid stools (12), poor nutritional status, medications (13), double incontinence (14), and higher PAT (Perineal Assessment Tool) score (7). However, the results of studies on risk factors for IAD are controversial due to geographical restrictions and sample size. Evidence on risk factors for IAD in critically ill patients is lacking. Therefore, this systematic review and meta-analysis was designed to identify risk factors for IAD. Based on that, nursing staff are more likely to pay attention to IAD in patients with specific risk factors and then provide them with appropriate care earlier to prevent the development of IAD.

## Methods

The protocol of this systematic review (register number: CRD42022353492) was registered in PROSPERO (http://www.crd.york.ac.uk/PROSPERO).

### Search strategy

In this meta-analysis, we searched the English electronic databases, Embase, PubMed, the Cochrane Library, and Web of Science, from their inception to July 2022. The terms searched were “ICU or intensive care or critically ill or critical care” and “incontinence-associated dermatitis or IAD or perineal dermatitis” and “risk factor or related factor or predictor”.

### Inclusion criteria

The following criteria were used to determine which studies would be included: (1) studies that addressed the risk factors for IAD in critically ill patients; (2) studies that were cohort studies or case-control studies; (3) studies published in English.

### Exclusion criteria

The following were the exclusion criteria: (1) case series, duplicate reports, reviews, and conference reports; (2) missing or abnormal data; (3) low quality of literature [Newcastle-Ottawa Scale (NOS) score < 6].

### Data extraction

Two authors independently screened the retrieved literature in line with the inclusion and exclusion criteria, and the following data information was extracted: name of the first author, country, study type, time of publication, basic characteristics of the included cases, and possible risk factors for IAD in critically ill patients. Discrepancies were discussed and solved by the researchers.

### Quality assessment

The Newcastle-Ottawa Scale (NOS) was used to evaluate the quality of the included literature (15), and the evaluation items include three aspects of population selection, comparability, and exposure evaluation. This scale included eight items with a score out of 9. Two researchers independently finished the quality assessment, if there is any dispute then a third researcher decides.

### Statistical analysis

Review Manager 5.4 was used for the statistical analysis. The heterogeneity of the included literature was assessed using *I*^2^. The fixed-effects model was used when *p* > 0.1 and *I*^2^ < 50%; otherwise, the random-effects model was used. One study was eliminated at a time in the sensitivity analysis. The OR and its 95% CI were calculated for count data, and differences were considered statistically significant at *p* < 0.05. When there were more than three studies included in the individual risk factor analysis, Egger's test was used to check for publication bias. The results showed *P* ≥ 0.05, suggesting that the included studies' publication bias was insignificant.

## Results

### Study selection

**Figure 1:**
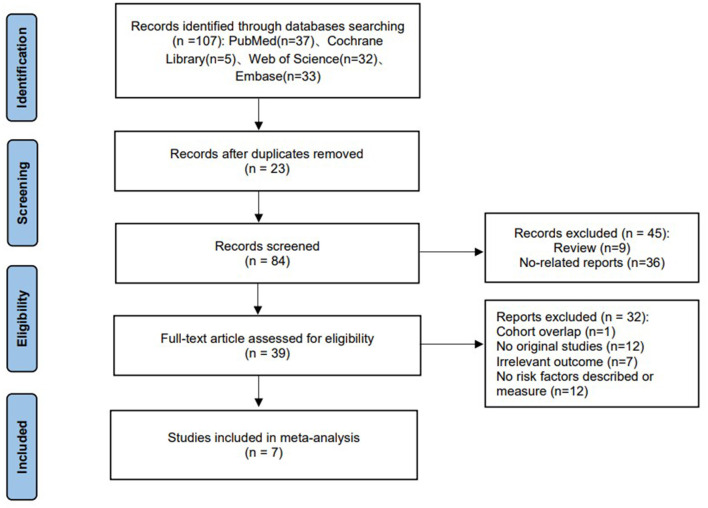
Study flow diagram.

There were 107 results from the literature search; most studies were excluded because they were not relevant to our study. 39 studies were then excluded because they did not meet the inclusion criteria after the full-text articles were reviewed. Finally, seven studies involving 1,238 recipients (432 cases and 806 controls) were included in this study. The study selection process is shown in Figure 1.

### Study characteristics

The specific characteristics of the studies included in the meta-analysis are presented in Table 1. These 7 studies were published from 2011 to 2019. They were conducted in Australia (5), Belgium (16), Brazil (17), China (7, 14, 18) USA (9). A total of 6 studies were cohort studies (5, 7, 9, 14, 17, 18) and 1 was case–control study (16). All the included studies scored ≥ 6 after being assessed by the NOS.

**Table 1:** Characteristics of studies included in the meta-analysis.

**Study**	**Year**	**Type of study**	**Country**	**N**	**Age (years)**	**Gender (%men)**	**Cases/controls**	**Quality assessment**
Bliss et al. (9)	2011	Cohort	USA	45	49.4 ± 18.5	76.0	16/29	7 points
Campbell et al. (5)	2018	Cohort	Australia	351	58.0	56.4	59/292	7 points
Chianca et al. (17)	2017	Cohort	Brazil	157	60.0 ± 17.0	45.9	32/125	8 points
Ma et al. (18)	2017	Cohort	China	104	63.09 ± 15.63	69.2	30/74	6 points
Van Damme et al. (16)	2018	Case-control	Belgium	206	65.1 ± 14.9	33.0	95/111	7 points
Wang et al. (14)	2018	Cohort	China	109	70.27 ± 15.31	62.4	26/83	7 points
Wei et al. (7)	2019	Cohort	China	266	64.18 ± 17.10	68.4	174/92	8 points

### Factors associated with incontinence-associated dermatitis

**Figure 2:**
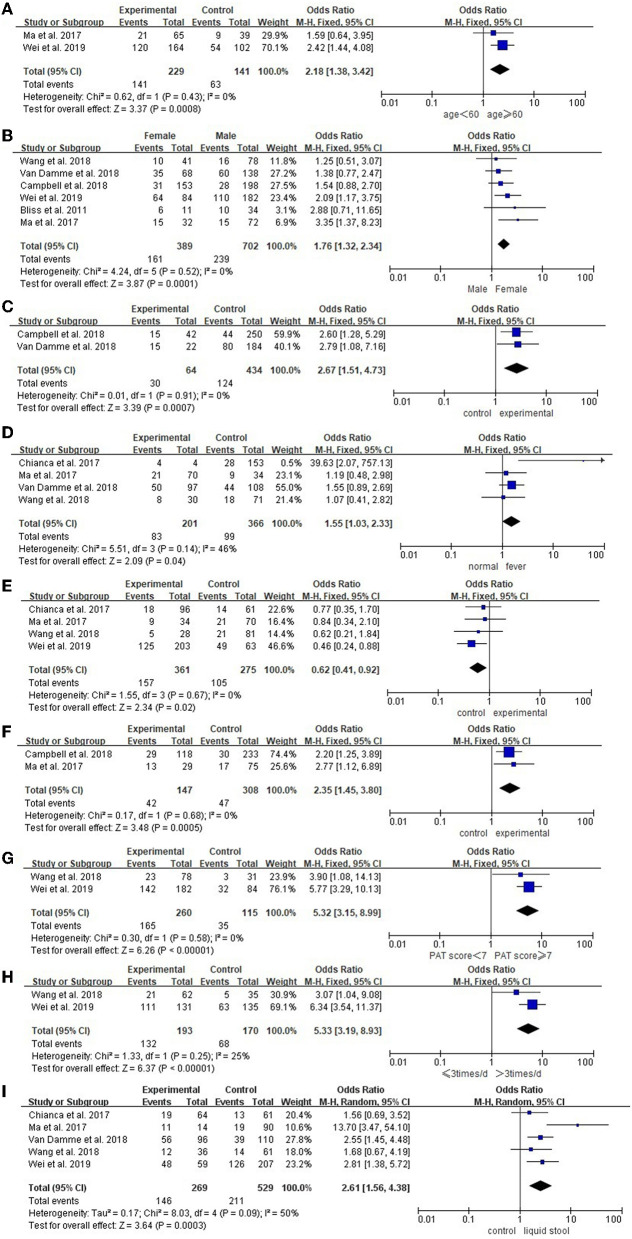
Forest plots of risk factors of IAD in critically ill patients. **(A)** Age; **(B)** Gender; **(C)** Dialysis; **(D)** Fever; **(E)** Sedative; **(F)** Vasoactive agent; **(G)** PAT score; **(H)** Frequency of bowel movement; **(I)** Liquid stool.

The meta-analysis showed that risk factors for IAD in critically ill patients included age ≥ 60 (OR = 2.18, 95% CI: 1.38~3.42), female sex (OR = 1.76, 95% CI: 1.32~2.34), dialysis (OR = 2.67, 95% CI: 1.51~4.73), fever (OR = 1.55, 95% CI: 1.03~2.33), vasoactive agent (OR = 2.35, 95% CI: 1.45~3.80), PAT (perineal assessment tool) score ≥ 7 (OR = 5.23, 95% CI: 3.15~8.99), frequency of bowel movement > 3 times/d (OR = 5.33, 95% CI: 3.19~8.93), and liquid stool (OR = 2.61, 95% CI: 1.56~4.38); sedative was concluded that a protective factor against IAD (OR = 0.62, 95% CI: 0.41~0.92) (Figure 2). There was no obvious correlation between risk factors and IAD, such as mechanical ventilation, antibiotic, enteral nutrition, albumin, diabetes, and urinary incontinence (Table 2).

**Table 2:** Meta-analysis of risk factors of incontinence-associated dermatitis in critically ill patients.

**Risk factors**	**Combination studies**	**OR (95%CI)**	**Z**	***P***	**Heterogeneity of study design**			**Analysis model**	**Egger's test**
					***Chi*** **^2^**	***P***	***I*** **^2^**		
Age	2	2.18 [1.38,3.42]	3.37	<0.001	0.62	0.43	0%	Fixed	NA
Gender	6	1.76 [1.32, 2.34]	3.87	<0.001	4.24	0.52	0%	Fixed	0.363
Mechanical ventilation	4	1.00 [0.33, 3.04]	0.00	1.00	24.20	<0.001	88%	Random	0.554
Antibiotic	3	1.35 [0.70, 2.62]	0.89	0.37	0.31	0.86	0%	Fixed	0.440
Enteral nutrition	4	1.18 [0.51, 2.72]	0.38	0.70	10.29	0.02	71%	Random	0.990
Dialysis	2	2.67 [1.51, 4.73]	3.39	<0.001	0.01	0.91	0%	Fixed	NA
Fever	4	1.55 [1.03, 2.33]	2.09	0.04	5.51	0.14	46%	Fixed	0.342
Albumin	3	1.34 [0.78, 2.28]	1.06	0.29	4.20	0.12	52%	Random	0.502
Diabetes	2	0.76 [0.51, 1.13]	1.34	0.18	0.46	0.50	0%	Fixed	NA
Sedative	4	0.62 [0.41, 0.92]	2.34	0.02	1.55	0.67	0%	Fixed	0.387
Vasoactive agent	2	2.35 [1.45, 3.80]	3.48	<0.001	0.17	0.68	0%	Fixed	NA
PAT score	2	5.32 [3.15, 8.99]	6.26	<0.001	0.30	0.58	0%	Fixed	NA
Urinary incontinence	2	1.60 [0.70, 3.65]	1.11	0.27	0.00	0.99	0%	Fixed	NA
Frequency of bowel movement	2	5.33 [3.19, 8.93]	6.37	<0.001	1.33	0.25	25%	Fixed	NA
Liquid stool	5	2.61 [1.56, 4.38]	3.64	<0.001	8.03	0.09	50%	Random	0.410

### Sensitivity analysis

Sensitivity analysis manifested that the result of the age, gender, mechanical ventilation, antibiotic, dialysis, diabetes, fever, sedative, vasoactive agent, PAT score, urinary incontinence, and frequency of bowel movement was stable. Nevertheless, “Campbell et al. (5)” had an influence on heterogeneity for enteral nutrition (*I*^2^ = 71% vs. *I*^2^ = 1%). Also, “Ma et al. (18)” was the major cause of heterogeneity for albumin (*I*^2^ = 52% vs. *I*^2^ = 0%) and liquid stool (*I*^2^ = 50% vs. *I*^2^ = 0%).

### Publication bias

Egger's test was conducted to evaluate potential publication bias (Table 2). The results revealed that most risk factors did not have a publication bias (*P* > 0.05).

## Discussion

IAD is a common skin injury in critically ill patients, which greatly affects the quality of life of patients (14). Therefore, identifying risk factors is essential for help preventing IAD. As a result, individuals at high risk should receive tailored prophylaxis. Several studies have reported potential risk factors for IAD among critically ill patients. The risk factors, however, have been inconsistent, possibly as a result of the various studies' use of various inclusion criteria or study designs. The objective of the current meta-analysis was to determine the risk factors for IAD in critically ill patients and provide the best evidence for clinical applications.

IAD was associated with the critically ill patient's general condition, our study showed that the risk of IAD in recipients with age ≥ 60 years was 2.18 times higher than the recipients with age < 60 years, which agreed with the findings of earlier research (19). The aging processes make the skin more prone to damage as people become older. These include the breakdown of collagen and connective tissue, which can reduce the flexibility of the skin (19). The epidermis thins and loses some of its flexibility as we age, cell turnover slows down, and skin fragility increases. The effect is that the skin is drier, more delicate, and vulnerable to damage from excrement (20). Older persons were at risk for IAD due to a high incontinence and skin aging incidence rate. On the other hand, due to their anatomical structure, females were more prone to IAD when urinary incontinence occurs when they were resting, specifically the region around the perineum, coccyx, and sacrum that is exposed to urine (21). The state of the perineal skin in female and elderly individuals needs to be given more consideration. The pediatric population is also the high-risk group for IAD, according to research, diaper dermatitis accounts for 1 in 5 pediatric dermatology visits, with IAD specifically being the most common presentation (22). However, there is a limited international consensus regarding the prevention of pediatric patients with IAD (23).

Our investigation also discovered that the risk of IAD increased with the fever which agrees with the studies of Demarre et al. (24). Fever patients have a higher rate of cell metabolism and an associated increase in energy demands, as well as skin hypoxia, accelerated sweat production brought on by fever, and decreased skin barrier function (24). Nursing staff should take timely and appropriate measures to lower the body temperature of febrile patients. Dialysis was a relevant factor in IAD, patients receiving dialysis were more likely to be malnourished (25). There should be more attention on dialysis patients' nutritional state. Compared to patients who do not use sedatives, patients who use sedatives have lower cognitive states, increasing their risk of developing IAD (17). However, our study found that sedative users had a lower risk of IAD. According to a study (26), critically ill patients who take sedatives require more care time, which enables patients to receive more skincare. The vasoactive agent has a close relationship with IAD occurrence, they may have an impact on tissue perfusion and exacerbate skin hypoxia (4). Therefore, nursing staff should pay more attention to patients' skin when applying vasoactive agents.

The probability of developing IAD was associated with the perineal environment. Patients with PAT ≥ 7 had a 5.32 times higher risk of developing IAD compared with those with PAT < 7 points, Li et al. (27) supported these findings and showed that 7.5 points was the cut point for identifying patients who were at a high risk of developing IAD. Patients with a PAT score higher than 7 need more active nursing intervention. The results of our investigation showed that the risk of developing IAD increased with bowel movement frequency, which was in agreement with earlier investigations (28). The more water the stool, the higher the risk of developing IAD. With a larger surface area in touch with the perineal skin and a greater density of bile salts and pancreatic lipase, liquid stools have a higher risk of harming the skin (29), which suggested nursing staff remove the feces promptly to prevent the occurrence of IAD. A meta-analysis indicated that using a skin cleanser to remove feces might be more effective than using soap and water (30).

This meta-analysis showed that mechanical ventilation, antibiotic, enteral nutrition, albumin, diabetes, and urinary incontinence were not the risk factors for IAD. This may be related to the small sample size in the literature. Further research is needed in the future.

This meta-analysis had several limitations. First, we only included English-language literature from four databases, there may not have been sufficient retrieval. Moreover, some risk factor indicators in this meta-analysis were not combined effectively because of the limited amount of literature, which might have impacted the results.

## Conclusion

Through this systematic review and meta-analysis, we have found a few risk factors for IAD in critically ill patients, which might provide a basis for clinical prevention. Future large-scale prospective cohort studies will be required to identify further risk factors for IAD in critically ill patients, which will be helpful for IAD care and prevention moving forward.

## Data availability statement

The original contributions presented in the study are included in the article/supplementary material, further inquiries can be directed to the corresponding author.

## Author contributions

HJ designed the study and wrote the first draft of the manuscript. HJ, HL, and JS verified data extraction and data analysis. QX and YL supervised the data acquisition, data analysis, and interpretation. LC reviewed the manuscript. All authors read and approved the final manuscript.

## References

[1] Gray M Black JM Baharestani MM Bliss DZ Colwell JC Goldberg M . Moisture-associated skin damage: overview and pathophysiology. J Wound Ostomy Continence Nurs. (2011) 38:233–41. 10.1097/WON.0b013e318215f798 21490547

[2] Chen Y Gao Y Zhang J Niu M Liu X Zhang Y Tian J. Quality and clinical applicability of recommendations for incontinence-associated dermatitis: a systematic review of guidelines and consensus statements. J Clin Nurs. (2022). 10.1111/jocn.16306 35411654

[3] Pather P Hines S. Best practice nursing care for ICU patients with incontinence-associated dermatitis and skin complications resulting from faecal incontinence and diarrhoea. Int J Evid Based Healthc. (2016) 14:15–23. 10.1097/XEB.0000000000000067 26735567

[4] Beeckman D A. decade of research on Incontinence-Associated Dermatitis (IAD): Evidence, knowledge gaps and next steps. J Tissue Viability. (2017) 26:47–56. 10.1016/j.jtv.2016.02.004 26949126

[5] Campbell JL Coyer FM Osborne SR. Incontinence-associated dermatitis: a cross-sectional prevalence study in the Australian acute care hospital setting. Int Wound J. (2016) 13:403–11. 10.1111/iwj.12322 24974872 PMC7949905

[6] Gray M Giuliano KK. Incontinence-associated dermatitis, characteristics and relationship to pressure injury: a multisite epidemiologic analysis. J Wound Ostomy Continence Nurs. (2018) 45:63–7. 10.1097/WON.0000000000000390 29300291 PMC5757660

[7] Wei L Bao Y Chai Q Zheng J Xu W. Determining risk factors to develop a predictive model of incontinence-associated dermatitis among critically ill patients with fecal incontinence: a prospective, quantitative study. Wound Manag Prev. (2019) 65:24–33. 30994472

[8] Valls-Matarín J Del Cotillo-Fuente M Ribal-Prior R Pujol-Vila M Sandalinas-Mulero I. Incidence of moisture-associated skin damage in an intensive care unit. Enferm Intensiva. (2017) 28:13–20. 10.1016/j.enfie.2017.03.005 28110903

[9] Bliss DZ Savik K Thorson MA Ehman SJ Lebak K Beilman G. Incontinence-associated dermatitis in critically ill adults: time to development, severity, and risk factors. J Wound Ostomy Continence Nurs. (2011) 38:433–45. 10.1097/WON.0b013e318220b703 21747261

[10] Beeckman D Van Lancker A Van Hecke A Verhaeghe S A. systematic review and meta-analysis of incontinence-associated dermatitis, incontinence, and moisture as risk factors for pressure ulcer development. Res Nurs Health. (2014) 37:204–18. 10.1002/nur.21593 24700170

[11] Bayón García C Binks R De Luca E Dierkes C Franci A Gallart E . Prevalence, management and clinical challenges associated with acute faecal incontinence in the ICU and critical care settings: the FIRST cross-sectional descriptive survey. Intensive Crit Care Nurs. (2012) 28:242–50. 10.1016/j.iccn.2012.01.005 22386584

[12] Johansen E Lind R Sjøbø B Petosic A. Moisture associated skin damage (MASD) in intensive care patients: a Norwegian point-prevalence study. Intensive Crit Care Nurs. (2020) 60:102889. 10.1016/j.iccn.2020.102889 32536519

[13] Coyer F Campbell J. Incontinence-associated dermatitis in the critically ill patient: an intensive care perspective. Nurs Crit Care. (2018) 23:198–206. 10.1111/nicc.12331 29266568

[14] Wang X Zhang Y Zhang X Zhao X Xian H. Incidence and risk factors of incontinence-associated dermatitis among patients in the intensive care unit. J Clin Nurs. (2018) 27:4150–7. 10.1111/jocn.14594 29964368

[15] Kim SY Park JE Lee YJ Seo H-J Sheen S-S Hahn S . Testing a tool for assessing the risk of bias for nonrandomized studies showed moderate reliability and promising validity. J Clin Epidemiol. (2013) 66:408–14. 10.1016/j.jclinepi.2012.09.016 23337781

[16] Van Damme N Clays E Verhaeghe S Van Hecke A Beeckman D. Independent risk factors for the development of incontinence-associated dermatitis (category 2) in critically ill patients with fecal incontinence: a cross-sectional observational study in 48 ICU units. Int J Nurs Stud. (2018) 81:30–9. 10.1016/j.ijnurstu.2018.01.014 29428583

[17] Chianca TC Goncales PC Salgado PO Machado BO Amorim GL Alcoforado CL. Incontinence-associated dermatitis: a cohort study in critically ill patients. Rev Gaucha Enferm. (2017) 37:e68075. 10.1590/1983-1447.2016.esp.68075 28380152

[18] Ma ZZ Song JY Wang M. Investigation and analysis on occurrence of incontinence-associated dermatitis of ICU patients with fecal incontinence. Int J Clin Exp Med. (2017) 10:7443–9.

[19] Lumbers M. How to manage incontinence-associated dermatitis in older adults. Br J Community Nurs. (2019) 24:332–7. 10.12968/bjcn.2019.24.7.332 31265344

[20] Beldon P. Incontinence-associated dermatitis: protecting the older person. Br J Nurs. (2012) 21:402–7. 10.12968/bjon.2012.21.7.402 22585017

[21] Sugama J Sanada H Shigeta Y Nakagami G Konya C. Efficacy of an improved absorbent pad on incontinence-associated dermatitis in older women: cluster randomized controlled trial. BMC Geriatrics. (2012) 12:22. 10.1186/1471-2318-12-22 22642800 PMC3426468

[22] Klunk C Domingues E Wiss K. An update on diaper dermatitis. Clin Dermatol. (2014) 32:477–87. 10.1016/j.clindermatol.2014.02.003 25017459

[23] Lim YSL Carville K. Prevention and management of incontinence-associated dermatitis in the pediatric population: an integrative review. J Wound Ostomy Continence Nurs. (2019) 46:30–7. 10.1097/WON.0000000000000490 30608338

[24] Demarre L Verhaeghe S Van Hecke A Clays E Grypdonck M Beeckman D. Factors predicting the development of pressure ulcers in an at-risk population who receive standardized preventive care: secondary analyses of a multicentre randomised controlled trial. J Adv Nurs. (2015) 71:391–403. 10.1111/jan.12497 25134858

[25] Yabe H Okada K Kono K Imoto Y Onoyama A Ito S Moriyama Y Kasuga H Ito Y. Exercise intolerance and malnutrition associated with all-cause mortality in elderly patients undergoing peritoneal dialysis: a single-center prospective cohort study. Int Urol Nephrol. (2022) 1–8. 10.1007/s11255-022-03446-4 36562903

[26] Dasta JF Kane-Gill S. Pharmacoeconomics of sedation in the ICU. Crit Care Clin. (2009) 25:571–83. 10.1016/j.ccc.2009.03.002 19576531

[27] Li Y-M Lee HH-C Lo Y-L Chao H-L. Perineal assessment tool (PAT-C): validation of a chinese language version and identification of a clinically validated cut point using ROC curve analysis. J Wound Ostomy Continence Nurs. (2019) 46:150–3. 10.1097/WON.0000000000000510 30844871

[28] Bliss DZ Savik K Harms S Fan Q Wyman JF. Prevalence and correlates of perineal dermatitis in nursing home residents. Nurs Res. (2006) 55:243–51. 10.1097/00006199-200607000-00004 16849976

[29] Woo KY Beeckman D Chakravarthy D. Management of moisture-associated skin damage: a scoping review. Adv Skin Wound Care. (2017) 30:494–501. 10.1097/01.ASW.0000525627.54569.da 29049257 PMC5657465

[30] Beeckman D Van Damme N Schoonhoven L Van Lancker A Kottner J Beele H . Interventions for preventing and treating incontinence-associated dermatitis in adults. Cochrane Database Syst Rev. (2016) 11:CD011627. 10.1002/14651858.CD011627 27841440 PMC6464993

